# Real-Time Telerehabilitation in Older Adults With Musculoskeletal Conditions: Systematic Review and Meta-analysis

**DOI:** 10.2196/36028

**Published:** 2022-09-01

**Authors:** Nathaphon Jirasakulsuk, Pattaridaporn Saengpromma, Santhanee Khruakhorn

**Affiliations:** 1 Department of Physical Therapy Faculty of Allied Health Sciences Thammasat University Pathum Thani Thailand

**Keywords:** telerehabilitation, internet-based intervention, older adults, physical performance, physical therapy

## Abstract

**Background:**

Real-time telerehabilitation (TR) is a new strategy for delivering rehabilitation interventions to older adults with musculoskeletal conditions, to provide continuity to conventional services and mitigate travel-related barriers.

**Objective:**

We aimed to examine the effectiveness of treatment delivered via real-time TR services compared to conventional services among older adults with musculoskeletal conditions, in terms of physical performance, treatment adherence, and cost-effectiveness.

**Methods:**

A literature search of randomized controlled trials (RCTs) published from January 2000 to April 2022 was conducted in six online databases: Cochrane Library, PubMed (ie, MEDLINE), PEDro, ClinicalKey, EBSCO, and ProQuest. The main eligibility criterion for articles was the use of real-time TR among older adults with musculoskeletal conditions to improve physical performance. Two reviewers screened 2108 abstracts and found 10 studies (n=851) that met the eligibility criteria. Quality assessment was based on version 2 of Cochrane’s risk-of-bias tool for RCTs, in order to assess the methodological quality of the selected articles. Results were pooled for meta-analyses, based on the primary outcome measures, and were reported as standardized mean differences (SMDs) with 95% CIs. A fixed model was used, and subgroup analysis was performed to check for possible factors influencing TR’s effectiveness based on different treatments, controls, and outcome measures.

**Results:**

The search and screening process identified 10 papers that collectively reported on three musculoskeletal conditions in older adults and three types of TR programs. Aggregate results suggested that real-time TR, compared to conventional treatment, was more effective at improving physical performance regarding balance (SMD 0.63, 95% CI 0.36-0.9; *I*^2^=58.5%). TR was slightly better than usual care at improving range of motion (SMD 0.28, 95% CI 0.1-0.46; *I*^2^=0%) and muscle strength (SMD 0.76, 95% CI 0.32-1.2; *I*^2^=59.60%), with moderate to large effects. Subgroup analyses suggested that real-time TR had medium to large effects favoring the use of smartphones or tablets (SMD 0.92, 95% CI 0.56-1.29; *I*^2^=45.8%), whereas the use of personal computers (SMD 0.25, 95% CI –0.16 to 0.66; *I*^2^=0%) had no effect on improving balance and was comparable to conventional treatment.

**Conclusions:**

We found that real-time TR improved physical performance in older adults with musculoskeletal conditions, with an effectiveness level equal to that of conventional face-to-face treatment. Therefore, real-time TR services may constitute an alternative strategy for the delivery of rehabilitation services to older adults with musculoskeletal conditions to improve their physical performance. We also observed that the ideal device for delivering TR is the smartphone. Results suggested that the use of smartphones for TR is driven by ease of use among older adults. We encourage future studies in areas related to rehabilitation in older adults, in addition to examination of physical performance outcomes, to gain additional knowledge about comprehensive care.

**Trial Registration:**

PROSPERO CRD42021287289; https://www.crd.york.ac.uk/prospero/display_record.php?RecordID=287289

## Introduction

Telerehabilitation (TR) was introduced to modify rehabilitation services that can be delivered to patients through interventions. It is currently used to increase the effectiveness of long-term treatment. There are various ways of receiving these interventions; technology helps in facilitating two-way communication via phone, video conferencing, and chat and health care apps. TR interventions have resulted in clinical outcomes being similar to or better than face-to-face (FTF) treatments; they are also in high compliance with home programs [1-4]. Previous systematic reviews evaluated TR for people with different health conditions in terms of feasibility, efficacy, and costs. The reviews supported the effectiveness of TR as an alternative to FTF interventions [5-8].

Musculoskeletal conditions affect 25% of the world’s population and are the leading cause of pain and disability [9]. This study defines musculoskeletal conditions as any diagnosed primary musculoskeletal condition, including those requiring operations. These disorders are more common among older adults [6], and their prevalence ranges from 5% to 74%, depending on the particular musculoskeletal disease. Older adults have been defined as people 60 years of age or above [10]. From a health care perspective, there is an increasing demand for health care among the older adult population; as such, health care providers recognize this burden on the health care system and have increased their awareness of the health and disability of this population. Consequently, there is a need for better rehabilitation services to address the current magnitude and impact of musculoskeletal conditions, as the number of patients grows.

It has been found that older adults who closely follow physiotherapy recommendations experience better treatment outcomes [11]. However, many older adult patients with geographical isolation or who lack local service availability continue to experience restrictions in appropriate and timely care as a result of increases in cost and wait times for orthopedic health services, as well as poor access to these services [12-14]. Older adults typically also have low adherence to home exercise programs [15]. This leads to a need for real-time interventions through, for example, the use of phone calls or video conferencing to deliver exercise information without a need to meet physiotherapists at the clinic. An increased awareness and understanding of different learning styles [16,17] and the emergence of new technologies have created opportunities for real-time TR instructions to be provided through a wider variety of formats; this could promote better adherence and, ultimately, better functional outcomes.

Recently, there have been many studies on the effectiveness of TR in the management of health conditions, such as stroke [18,19], chronic obstructive pulmonary disease (COPD) [20,21], and heart disease [22,23]. However, those studies rarely explored the management of musculoskeletal conditions among older adults through TR. This became a hindrance to the implementation of TR as another way to deliver health care services. Previous systematic reviews about musculoskeletal conditions in older adults [6,24,25] examined studies with different conclusions. The aim of this systematic review was to (1) determine whether older adults with musculoskeletal conditions can improve physical performance via real-time TR and whether the results are effective compared with conventional services and (2) compare adherence to and cost-effectiveness of real-time TR with that of conventional treatments.

## Methods

We systematically conducted and reported results of our review in accordance with the PRISMA (Preferred Reporting Items for Systematic Reviews and Meta-Analyses) guidelines [26]. This review was registered at PROSPERO (International prospective register of systematic reviews; CRD42021287289).

### Search Strategy

An electronic literature search was conducted on April 10, 2022, in six online databases: Cochrane Library, PubMed (ie, MEDLINE), PEDro, ClinicalKey, EBSCO, and ProQuest. The literature search was limited to articles written in English and Thai languages that were published from January 1, 2000, to the date of the search. Studies in other languages were not compiled. We used a search strategy that combines Medical Subject Headings with free keywords and connected them with Boolean conjunctions (ie, OR and AND). Keywords included “real-time telerehabilitation,” “real-time internet-based,” and “remote exercise.” The search strategy and keywords were developed through discussion and peer review between two authors (NJ and PS); keywords included specific search terms related to research objectives. Details of the search strategy and keywords are given in Multimedia Appendix 1.

### Eligibility Criteria

In this systematic review, we included only randomized controlled trials (RCTs) that studied the effects of real-time TR interventions on the physical performance of older adults with musculoskeletal conditions. The specific eligibility criteria used for selecting studies were established; these were based on the PICO (population, intervention, comparison, and outcome) framework (Table 1 [27]).

**Table 1:** Eligibility criteria for inclusion of articles in the study.

Criterion type	Inclusion criteria	Exclusion criteria
Study design	Randomized controlled trials	Systematic reviews Case studies Cross-sectional studies
Population	Community-dwelling older adults with musculoskeletal conditions, aged 60 years and over, receiving exercise interventions for health conditions	People not actively seeking or accessing health care Services targeting recipients of health promotion measures, screening, and so on
Intervention	Physical therapy exercise instructions provided using real-time TR^a^; definition of real-time TR was quoted from a previous study [27] describing the interventions as follows: provided by means of any kind of technological device allowing for health care professional–patient interaction online, provided by health care professionals or caregivers through remote supervision, and including at least one specific intervention targeted to rehabilitation (eg, teletraining, TR, telehealth, and internet based)	General information not tailored or selected specifically for individual patients Telemedicine using multimedia approaches, with an intention for patient action or behavior change
Comparison	Comparators included either usual physical therapy rehabilitation interventions, which were provided in person in a hospital or institution setting; educational interventions; or no specific interventions	N/A^b^
Outcome	Any kind of physical function or motor performance outcome (eg, mobility, balance, strength, and walking), adherence outcome (eg, complete rate), or cost-effectiveness outcome	Clinician outcomes Service-level outcomes Questionnaire results Self-report results

^a^TR: telerehabilitation.

^b^N/A: not applicable; the comparison criterion type did not have any exclusion criteria.

### Study Selection

In a standardized blinded manner, two authors (NJ and PS) independently identified potentially relevant papers and performed eligibility assessments. This process was divided into two phases. First, titles and abstracts were screened independently by two reviewers (NJ and PS). They assessed the relevance of each article and rated each one as definitely relevant, possibly relevant, or not relevant. Second, they screened the full text of the articles that had been judged in the first phase as definitely relevant or possibly relevant, and they made a final judgement on the articles as relevant or not relevant. In both phases, disagreement between the two reviewers was resolved through discussion until consensus was reached or through consultation with a third reviewer (SK). To assess the degree of agreement between authors, we calculated κ statistics for both phases. As part of our calculations, categories of definitely relevant and possibly relevant in the first phase were merged into the category definitely or possibly relevant. ^P^<.05 was considered statistically significant.

### Data Extraction

Extracted information for each article in this study included authors, publication year, study setting (ie, country or region), sample characteristics (eg, age, gender, and medical conditions), duration of study, description of interventions for both control and experimental groups (eg, information and communications technology [ICT] devices and platforms, intervention formula, presence or absence of in-person intervention during TR, compared intervention, and effects), outcome data from both processes (eg, intervention completion rate, reasons for withdrawal from the intervention, and adverse events during the intervention), and patient outcomes (eg, impairment, activities, and participation). Data extraction was completed by one reviewer (NJ) and checked for accuracy by the second reviewer (PS).

### Quality Assessment

Risk of bias was assessed using version 2 of Cochrane’s risk-of-bias tool (RoB 2) for randomized trials [28]. Two reviewers (NJ and PS) assessed all five domains independently. The domains are as follows: (1) bias arising from the randomization process, (2) bias due to deviations from intended interventions, (3) bias due to missing outcome data, (4) bias in measurement of the outcome, and (5) bias in selection of the reported result. Each domain contains several signaling questions for assigning one of three risk-of-bias levels to each domain: low risk of bias, some concerns, or high risk of bias. These assessments allow the assessor to judge the overall risk of bias in each trial. Lower-quality articles were not excluded from the meta-analysis.

### Statistical Analysis

Continuous data are presented as the mean difference or standardized mean difference (SMD) with 95% CI, whereas dichotomous data are presented as the risk ratio with 95% CI. If the studies did not adjust for clustering, we attempted to adjust their standard errors using the methods described in the Cochrane Handbook for Systematic Reviews of Interventions [29].

The *I*^2^ test was used to identify heterogeneity: homogeneity was set at *I*^2^<50%. The data were pooled by a fixed-effects model if eligible studies were included. In addition, Forest plots for the meta-analysis were conducted if more than 2 eligible RCTs were included. We conducted a sensitivity meta-analysis that was restricted to recently published (ie, in 2000 or later) RCTs with an overall low risk of bias (ie, low risk of bias in all 10 criteria). Otherwise, *I*^2^>50% was regarded as having substantial heterogeneity. Under such situations, a fixed model was used, and subgroup analysis was conducted to check for any possible reasons that could have caused substantial heterogeneity based on the different treatments, controls, and outcome measurements. After subgroup analysis, if the heterogeneity was still significant, a narrative summary was presented instead of pooled data and a meta-analysis.

Additionally, sensitivity analyses were also performed to check the robustness of the pooled results, depending on the different methodological qualities, and the statistical models. Furthermore, funnel plots were created, and the Begger test was carried out to identify any reporting biases if a sufficient number of eligible studies were included in the study.

## Results

### Study Selection

**Figure 1:**
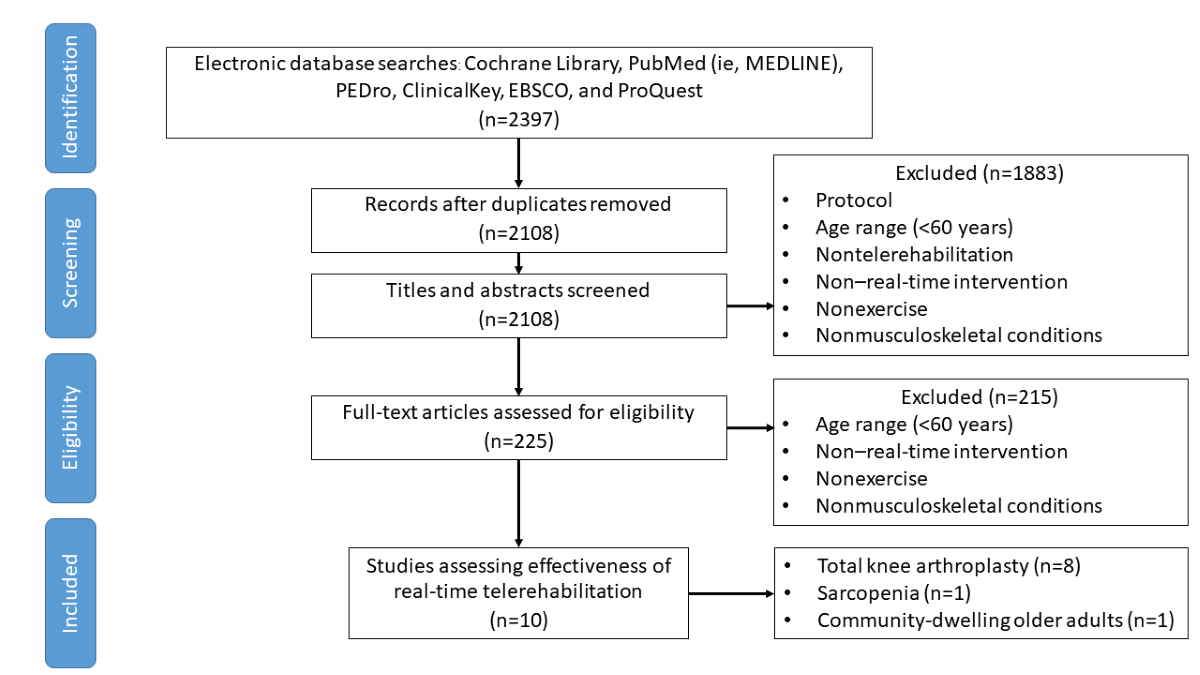
Flow of studies through the review.

The study selection process is shown in Figure 1. This was carried out through a literature search and identification of 2395 articles without duplication. The screening categorized 225 of the articles as being definitely relevant or potentially relevant, whereas the remainder were not considered relevant in the first phase. After screening the full text of 225 articles in the second phase, the reviewers found that 10 studies were eligible for inclusion in this systematic review. κ statistics for judgement in the first and second phases were 0.619 (^P^<.001) and 0.667 (^P^<.001), respectively. All conflicts regarding the judgments were reconciled through negotiations between the authors.

### Characteristics of the Studies

The 10 eligible RCTs are summarized in Table 2 [30-39]. All studies were conducted in South Korea, Canada, Portugal, the United States, and Australia. The participants’ health conditions, sample sizes, and sex ratios varied widely across the studies. Health conditions included patients with pre- and postoperative total knee arthroplasty (TKA) or total hip arthroplasty as well as community-dwelling older adults with sarcopenia. The total sample size was 851 participants, of which 351 (41.2%) were males. The ICT devices used in real-time TR were smartphones and personal computers. Out of 10 studies, 7 (70%) had developed a specific platform to administer their real-time TR; 2 (20%) used Skype, a free telecommunications app; and 1 (10%) used video conferencing.

The main components of the real-time TR interventions from the 10 studies included exercise regimens that varied across the studies: resistance exercise, combination resistance and range of motion (ROM) exercise, ROM exercise, and balance program exercise. Out of 10 studies, 2 (20%) included an educational intervention about self-management during real-time TR.

The interventions that were compared in the studies included in-clinic physical therapy, home visit rehabilitation, home exercise programs, education for general health, and nutrition. Because of the heterogeneity in the study characteristics, it was not appropriate to carry out a meta-analysis. Thus, a narrative analysis of the 7 eligible studies was conducted.

**Table 2:** Characteristics of the 10 eligible randomized controlled trials.

Author, year	Population			Intervention				Outcome report
	Size, n	Mean age (years)	Device		Platform	Type of intervention		
An et al, 2021 [30]	36	70.51	Smartphone		Video conferencing	Preoperative rehabilitation of TKA^a^	Quadriceps strength ROM^b^ of knee flexion Timed Up and Go test	
Doiron-Cadrin et al, 2020 [31]	34	65.97	Smartphone or tablet		Skype	Preoperative rehabilitation of TKA or THA^c^	Timed Up and Go test Stair test	
Fernando et al, 2018 [32]	59	68.65	Smartphone or tablet		Specially developed platform	Postoperative rehabilitation of TKA	ROM of knee flexion Timed Up and Go test	
Hong et al, 2017 [33]	23	81.85	Personal computer		Skype	Resistance and balance exercises	Timed Up and Go test Chair stand test	
Prvu Bettger et al, 2020 [34]	287	65.25	Personal computer		Specially developed platform	Postoperative rehabilitation of TKA	Total cost ROM of knee flexion	
Russell et al, 2003 [35]	21	67	Personal computer		Specially developed platform	Postoperative rehabilitation of TKA	ROM of knee flexion Timed Up and Go test Knee extensor strength	
Russell et al, 2011 [36]	65	67.9	Personal computer		Specially developed platform	Postoperative rehabilitation of TKA	ROM of knee flexion Timed Up and Go test Knee extensor strength	
Sparrow et al, 2011 [37]	100	71	Personal computer		Specially developed platform	Resistance exercise program	Knee extensor strength Single-leg stance	
Tousignant et al, 2011 [38]	41	66	Personal computer		Specially developed platform	Postoperative rehabilitation of TKA	ROM of knee flexion Berg Balance Scale 30-second chair stand test	
Tousignant et al, 2015 [39]	197	66	Personal computer		Specially developed platform	Postoperative rehabilitation of TKA	Total cost Cost per time	

^a^TKA: total knee arthroplasty.

^b^ROM: range of motion.

^c^THA: total hip arthroplasty.

### Risk-of-Bias Assessment

**Figure 2:**
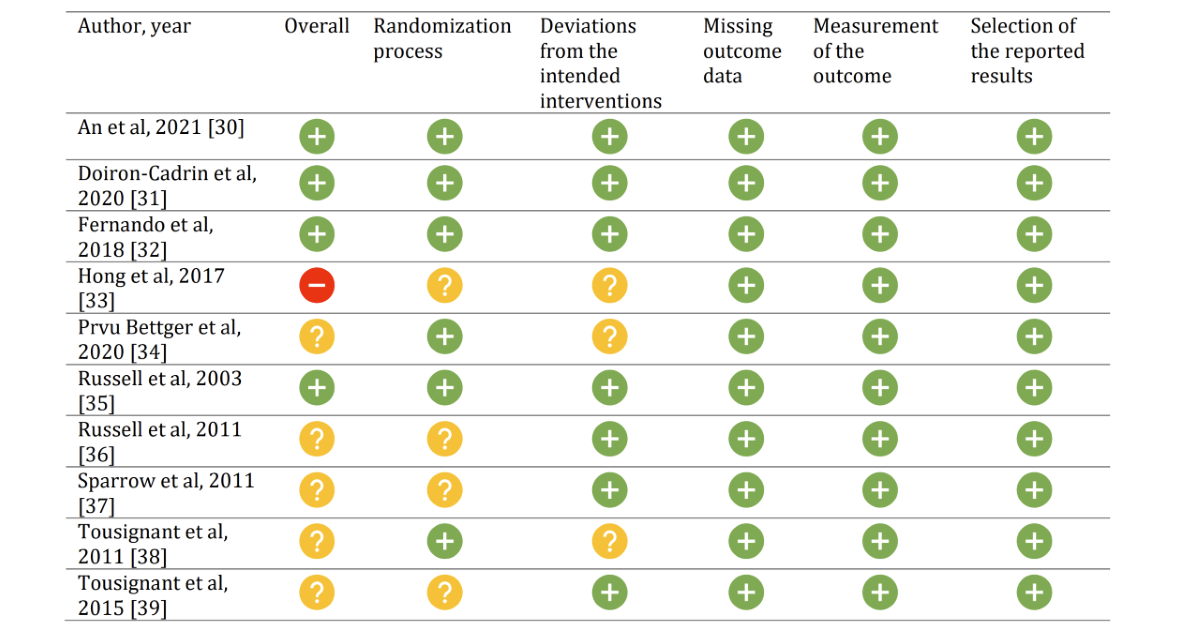
Summary of the the risk-of-bias assessment. A red circle with a minus sign indicates a high risk of bias, a yellow circle with a question mark indicates there are some concerns, and a green circle with a plus sign indicates a low risk of bias.

The results of the risk-of-bias assessment, based on the RoB 2, are summarized in Figure 2. The risk of bias in 1 study was high, 5 studies had some concerns, and 4 studies were categorized as having a low risk of bias. Regarding the risk of bias caused by the randomization process (domain 1), 4 studies used a nonconcealment approach, and the baseline characteristics were different between groups because they may have had some selection bias. Thus, regarding domain 1, most studies were assessed as having a risk of bias of some concern. The risk of bias due to deviations from intended interventions (domain 2) was judged to be low in 7 studies, which were assessed as having a risk of bias of some concern. Regarding the risk of bias due to missing outcome data (domain 3), outcome measurement (domain 4), and selection of the reported results (domain 5), all studies were judged as having a low risk of bias.

### Effects of Real-Time Telerehabilitation

#### Range of Motion

**Figure 3:**
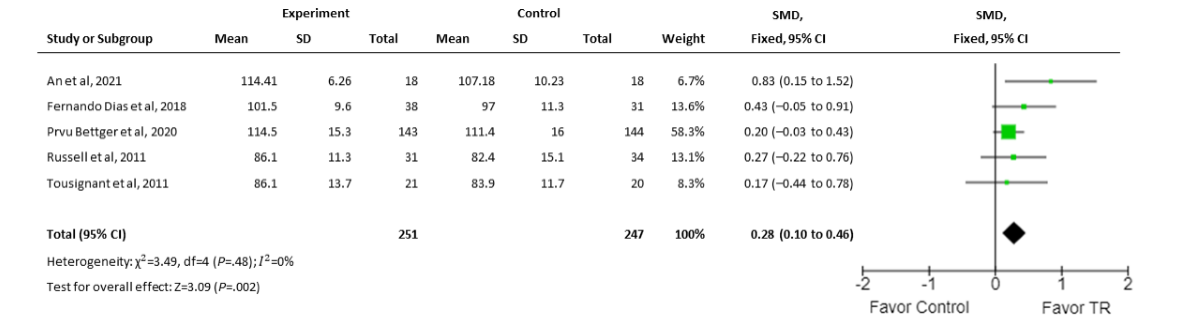
Meta-analysis comparing the effect of real-time telerehabilitation on range of motion of knee flexion following interventions for all conditions. SMD: standardized mean difference; TR: telerehabilitation.

The ROM of knee flexion was assessed in only 5 trials, as their primary outcomes were able to be pooled [30,32,34,36,38]. Data from only 498 participants were able to be pooled due to insufficient data in the ROM of knee flexion. Aggregate results showed a small effect in favor of real-time TR as compared to conventional service (SMD 0.28, 95% CI 0.1-0.46; *I*^2^=0%; Figure 3).

Subgroup analyses were conducted for specific musculoskeletal conditions and type of TR medium (ie, personal computer, smartphone, tablet, and platform). For rehabilitation of postoperative TKA, the pooled results of 4 trials [32,34,36,38] suggested that TR interventions were less favorable (SMD 0.24, 95% CI 0.06-0.42; *I*^2^=0%); while 3 RCTs had a risk of bias of some concern [34,36,38], they did not favor real-time TR for the improvement of ROM of knee flexion following postoperative TKA (SMD 0.21, 95% CI 0.01-0.4; *I*^2^=0%).

Regarding the intervention medium of real-time TR, 2 trials [30,32] that used smartphones or tablets showed a moderate effect favoring TR (SMD 0.56, 95% CI 0.18-0.95; *I*^2^=0%), whereas 3 studies that used personal computers with specific software [34,36,38] yielded a small effect in favor of TR (SMD 0.21, 95% CI 0.01-0.4; *I*^2^=0%).

#### Muscle Strength

Data from 3 trials assessing muscle strength as a primary outcome were able to be pooled. One trial used knee extensor strength [30], whereas 2 trials presented physical function data in the form of lower-limb strength from the sit-to-stand test [33] or the stair test [31]. Aggregate results of substantial statistical heterogeneity suggested that real-time TR had more moderate to large effects favoring the TR intervention as compared to usual care (SMD 0.76, 95% CI 0.32-1.2; *I*^2^=59.60%).

#### Balance

**Figure 4:**
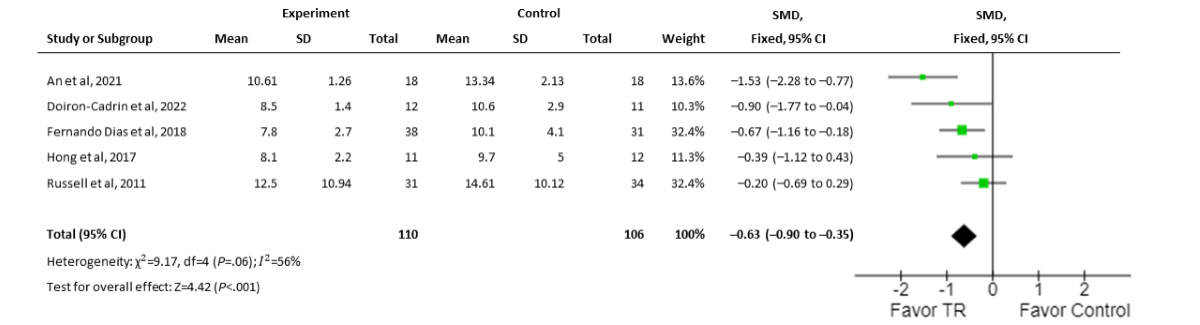
Meta-analysis comparing the effect of real-time telerehabilitation on balance following interventions for all conditions. SMD: standardized mean difference; TR: telerehabilitation.

In total, 5 trials assessed balance using the Timed Up and Go (TUG) test as a primary outcome. Data from only 216 participants were able to be pooled due to insufficient data. Aggregate results showed a moderate effect that favored real-time TR for the improvement of TUG test results (SMD 0.63, 95% CI 0.36-0.9; *I*^2^=58.5%; Figure 4) [30-33,36]. Meanwhile, 3 RCTs with a low risk of bias [30-32] had large effects that favored real-time TR (SMD 0.92, 95% CI 0.56-1.29; *I*^2^=45.8%).

Subgroup analyses further suggested that real-time TR interventions with medium to large effects favoring smartphones or tablets (SMD 0.92, 95% CI 0.56-1.29; *I*^2^=45.8%) [30-32] or personal computers (SMD 0.25, 95% CI –0.16 to 0.66; *I*^2^=0%) [33,36] have an equal effect on improvement of balance.

Finally, results from 4 trials that examined rehabilitation following preoperative TKA [30,31] or postoperative TKA (SMD 0.43, 95% CI 0.09-0.77; *I*^2^=45.4%) [32,36] specifically showed that TR was more favorable than usual rehabilitation care.

### Feasibility and Acceptance of Real-Time Telerehabilitation

Completion rates, reasons for withdrawal, and adverse events are shown in Table S1 in Multimedia Appendix 2. Completion rates were reported in all studies: mean 91.09% (SD 5.77%; range 85%-100%) in the experimental group and 94.89% (SD 5.18%; range 83.3%-100%) in the control group. Most studies described the reasons for withdrawal, including dropout, lack of follow-up, and hospitalization as a result of other diseases. However, these reasons were not described clearly in all studies. In total, 3 studies [31,32,37] reported that adverse events occurred during real-time TR, whereas the other 7 studies did not specifically describe adverse events.

Only 2 studies reported cost-effectiveness analysis, which evaluated the total costs of rehabilitation in older adults with osteoarthritis of the knee in the postoperative phase. Total costs of real-time TR (mean US $1502.98, SD $278.98) and usual care (mean US $3006.89, SD $1519.89) had moderate effects favoring real-time TR (SMD 0.69, 95% CI 0.51-0.88; *I*^2^=0%).

### Potential Contributing Factors to Feasibility

We summarized the data from factors potentially contributing to safety and feasibility based on treatments via video conference, as seen in Table S1 in Multimedia Appendix 2. For safety measures in 2 studies [33,37] in which community-dwelling older adults were recruited, data regarding the rate of perceived exertion were collected from a tolerance exercise program; the 2 studies also used the patients’ own records of pain, falls, and readmission to hospitals. Patients were accompanied by a caregiver when giving reports during the postoperative TKA treatment period. Finally, we found that 6 studies set minimum internet speeds ranging from 18 kbps to 10 Mbps, whereas 4 studies did not report this information.

## Discussion

### Principal Findings

The main finding of this study was that, following noninferiority analysis, one particular treatment is not inferior to the current standard treatment for a particular health condition [40]. Post hoc noninferiority analysis was undertaken to compare results between cohorts from the examined interventions when sufficient data were found for subgroup analyses. Results of the noninferiority analysis supports a conclusion that physiotherapy exercises for the TKA population via real-time TR is equivalent and not inferior to FTF care.

This study is the first systematic review that focuses on real-time TR services using phone calls or video conferencing for home-based exercising to improve physical performance and adherence to treatment in older adults with musculoskeletal conditions. These results showed that TR had similar or better effects, as compared to usual care, on older adults’ physical performance, including balancing ability, strength, and ROM. Furthermore, when compared to conventional care, small to moderate, but significant (^P^<.001), effects could be seen in favor of real-time TR, suggesting that real-time TR is superior to conventional services with respect to physical performance. Subgroup meta-analyses in this review showed small statistical heterogeneity across the studies due to trials that used smartphones or tablets (*I*^2^=0%). However, in those studies that only provided real-time TR treatment via personal computer or videoconferencing software, real-time TR still produced favorable outcomes, albeit to a small extent, as compared to FTF care following the intervention (*I*^2^=0%). Regardless of the musculoskeletal condition or by which medium the real-time TR was delivered, improvements in balance were also seen to be comparable between cohorts.

The primary findings in this review were similar to those of previous systematic reviews that reported positive benefits in patients following TKA [8], cardiac disease [3,41], and COPD [42] among community-dwelling older adults [43]. Articles from a systematic review and meta-analysis [8] on TR for TKA reported strong positive effects from a TR program (mean difference 1.14, 95% CI –0.61 to 2.89; *I*^2^=0%) and improvement in active knee flexion status that were similar to those among patients with improving health conditions in the conventional therapy group. TR seems to be a practical alternative to conventional FTF rehabilitation therapy in patients who underwent TKA. Similarly, a previous systematic review [6] aimed to examine TR for musculoskeletal conditions and evaluated the effectiveness of TR as compared to usual care in improving physical function and disability; a moderate effect was observed in favor of TR (SMD 0.45, 95% CI 0.20-0.70; *I*^2^=56%). In this review, only the selected primary outcome measures were pooled to estimate the effects of treatment interventions, in which the TUG test served as a validated assessment tool that was simple, quick, and able to be applied in a short time to measure physical performance in a TKA population. Therefore, improvement in physical function has been added to the growing body of evidence for the efficacy of real-time TR in older adults with musculoskeletal conditions.

In our review of adherence data across the selected studies, the intervention completion rate was available in all of them, with adherence reported to be 91% (range 85%-100%) in the experimental group. This figure is comparable with previously reported completion rates of real-time TR among older adults with COPD in Australia (95%) [44], those with hemiplegia in China (96%) [45], those with heart failure in Australia (95%) [46], and those suffering from peripheral artery disease in the United States (85%) [47]. However, a direct comparison of findings between studies is difficult due to discrepancies across the countries, participants’ conditions, and intervention regimens. Our findings imply that real-time TR interventions might constitute an acceptable rehabilitation strategy for older adults.

In contrast, there was less emphasis on other reporting regarding adherence, such as specific reasons for withdrawal from interventions and adverse events during interventions. In-depth interviews in a previous study [48] described specific reasons for withdrawal from their TR service among older adults, such as “one person withdrew because of the need to train at the local center” and “one person withdrew [from] the program after finding the technology too difficult to use the computer and IT platform.” These descriptions allow readers the opportunity to vary programs in order to simulate adherence or to design a program and judge the appropriateness of the intervention in order to make it more feasible for their clinical practice or research project. Future studies documenting this relevant information can improve real-time TR services.

### Comparison to Prior Work

Although the effectiveness of real-time TR among older adults with musculoskeletal conditions is apparent, findings from this review still need to be supported by additional studies. Thus, the interpretation of our findings require careful consideration. The poor methodological quality and heterogeneity in telehealth-related studies were also reported in previous systematic reviews [1,27,41,49]. A previous systematic review [43] insisted that there is a critical need for high-quality studies investigating the impact of TR interventions in older adults. Consequently, it is crucial that these issues be taken into consideration when further studies are conducted.

### Potential Future Directions

This study showed that the technologies used during real-time TR interventions varied across the included studies. In the examined studies, we observed variations in the health conditions of older adults, various kinds of technologies being used, and specific trends.

First, we observed that an app’s ease of use was important during real-time TR services among older adults with a health condition. Previous studies [50,51] found that interactive app use during individual interviews helped identify content for creating a prototype before designing a mobile health (mHealth) app. Thus, mHealth apps that are used for TR focus on user characteristics.

Second, health care providers are able to motivate and increase the self-confidence of older adults during real-time TR services. Previous studies that conducted in-depth interviews with patients found that motivational techniques, including giving feedback to patients regarding exercise during the intervention, were important for helping patients improve [52,53]. If a physiotherapist cannot give older adult patients clear and sufficient advice regarding their health conditions in order to improve their physical performance, the patients could lose their self-confidence and treatments could become ineffective. On the other hand, effective communication can enhance participants’ confidence and ensure positive outcomes from the treatment programs.

Finally, based on our findings, there was a moderate effect with a significant difference (SMD 0.69, 95% CI 0.51-0.88; *I*^2^=0%) on cost-effectiveness as a result of real-time TR as compared to usual care. It is reasonable to believe that the costs of real-time TR interventions can be lower than those incurred from conventional treatments with FTF communication between physiotherapists and patients [34,39]. The cost-effectiveness measure is an important factor for the treatment of patients, and it can vary depending on patients’ conditions during real-time TR interventions.

### Limitations

There were four limitations in our study. First, we had limited access to research databases and articles in different languages, preventing us from reviewing some research studies. Other research databases, such as Embase and ScienceDirect, were not used due to accessibility issues. Moreover, our review did not cover articles written in other languages. This selection bias may seriously have impacted our results and must be acknowledged when interpreting them.

Second, the focus of our review was limited to the outcome measures of physical performance and intervention adherence. Furthermore, other subjective outcome measures, such as pain scales, activities of daily living, quality of life, and feasibility indexes, should also be evaluated in future studies.

Third, caution was taken when we made conclusions about the overall effects of the management of musculoskeletal conditions. Because almost all of the trials examined interventions that followed common orthopedic surgery procedures in areas where access to conventional FTF interventions are limited, TR is an alternative treatment that could provide patients with sufficient availability of health care services.

Finally, as mentioned earlier, the number of selected studies with a low risk of bias was small, so real-time TR interventions must have high-quality methodologies to ensure that their effectiveness and results can be generalized to various treatments at clinics.

### Conclusions

This study showed that there is strong evidence to conclude that TR-based physiotherapy interventions are effective in improving physical performance among older adults with musculoskeletal conditions; in addition, treatment outcomes from TR can be as successful as those from conventional FTF treatment. This is the first systematic review that evaluated the effects of real-time TR in older adults with musculoskeletal conditions; our results indicated that real-time TR services can potentially constitute an alternative strategy for the delivery of rehabilitation services in patients with musculoskeletal conditions. Future rigorous clinical trials are warranted in order to formally establish the efficacy of TR in the management of specific musculoskeletal conditions.

## Appendix

Multimedia Appendix 1 Search strategy.

Multimedia Appendix 2 Summary of completion rates, reasons for withdrawal, and adverse events in the included studies.

## Abbreviations

COPD: chronic obstructive pulmonary disease
FTF: face-to-face
ICT: information and communications technology
mHealth: mobile health
PICO: population, intervention, comparison, and outcome
PROSPERO: International prospective register of systematic reviews
RCT: randomized controlled trial
RoB 2: version 2 of Cochrane’s risk-of-bias tool
ROM: range of motion
SMD: standardized mean difference
TKA: total knee arthroplasty
TR: telerehabilitation
TUG: Timed Up and Go
