# Supplementary material for: Real-Time Telerehabilitation in Older Adults With Musculoskeletal Conditions: Systematic Review and Meta-analysis
Source: JMIR Rehabil Assist Technol. 2022 Sep 1;9(3):e36028. doi: 10.2196/36028 (PMC9478822; doi:10.2196/36028)
Supplement: Multimedia Appendix 1 [file rehab_v9i3e36028_app1.pdf]

1. Search terms and search strategies in Cochrane

#1 Title Abstract Keyword: “Real-time telerehabilitation”
#2 Title Abstract Keyword: “Physical therapy”
#3 Publication Type: “Randomized control trial”
#4 #1 AND #2 AND #3

#1 Title Abstract Keyword: “Real-time internet based”
#2 Title Abstract Keyword: “Physical therapy”
#3 Publication Type: “Randomized control trial”
#4 #1 AND #2 AND #3

#1 Title Abstract Keyword: “Telerehabilitation”
#2 Title Abstract Keyword: “Physical therapy”
#3 Publication Type: “Randomized control trial”
#4 #1 AND #2 AND #3

#1 Title Abstract Keyword: "Internet based"
#2 Title Abstract Keyword: “Physical therapy”
#3 Publication Type: “Randomized control trial”
#4 #1 AND #2 AND #3

#1 Title Abstract Keyword: "Remote rehabilitation"
#2 Title Abstract Keyword: “Physical therapy”
#3 Publication Type: “Randomized control trial”
#4 #1 AND #2 AND #3

#1 Title Abstract Keyword: "Telehealth"
#2 Title Abstract Keyword: “Physical therapy”
#3 Publication Type: “Randomized control trial”
#4 #1 AND #2 AND #3

#1 Title Abstract Keyword: "Telemedicine"
#2 Title Abstract Keyword: “Physical therapy”
#3 Publication Type: “Randomized control trial”
#4 #1 AND #2 AND #3

#1 Title Abstract Keyword: "Mobile health"
#2 Title Abstract Keyword: “Physical therapy”
#3 Publication Type: “Randomized control trial”
#4 #1 AND #2 AND #3

#1 Title Abstract Keyword: "Mhealth"
#2 Title Abstract Keyword: “Physical therapy”

#3 Publication Type: “Randomized control trial”
#4 #1 AND #2 AND #3

#1 Title Abstract Keyword: "ehealth"
#2 Title Abstract Keyword: “Physical therapy”
#3 Publication Type: “Randomized control trial”
#4 #1 AND #2 AND #3

2. Search terms and search strategies in PubMed/MEDLINE

#1(Study design, publication year, language)
"Randomized Controlled trial"[Publication Type] AND 2000/1/1:3000/12/31[Date Publication]) AND ("English"[Language])

#2(Real-time Telerehabilitation)
"Real-time Telerehabilitation"[MeSH Terms] OR "Real-time internet based"[MeSH Terms] OR "Real-time Telerehabilitation"[Title/Abstract] OR "Real-time internet based"[Title/Abstract] OR "Realtime Telerehabilitation"[Title/Abstract] OR "Realtime internet based"[Title/Abstract] OR "Telerehabilitation"[Title/Abstract] OR "internet based intervention"[Title/Abstract] OR "Remote rehabilitation"[Title/Abstract] OR "Telehealth"[Title/Abstract] OR "Telemedicine"[Title/Abstract] OR "mobile health"[Title/Abstract] OR "mhealth"[Title/Abstract] OR "ehealth"[Title/Abstract] OR "social network service"[Title/Abstract]

#3(Physical Therapy)
"Physical Therapy"[All Fields] OR "Physiotherapist"[All Fields] OR " Physiotherapist"[All Fields]

#4
#1 AND #2 AND #3
124

3. Search terms and search strategies in Pedro

#1 Abstract & Title: “Real-time telerehabilitation”
#2 Method “Clinical trial”
#3 Published Since: 2000
#4 #1 AND #2 AND #3

#1 Abstract & Title: “Real-time internet based”
#2 Method “Clinical trial”
#3 Published Since: 2000
#4 #1 AND #2 AND #3

#1 Abstract & Title: "Telerehabilitation"
#2 Method "Clinical trial"
#3 Published Since: 2000
#4 #1 AND #2 AND #3

#1 Abstract & Title: "Internet based intervention"
#2 Method "Clinical trial"
#3 Published Since: 2000
#4 #1 AND #2 AND #3

#1 Abstract & Title: "Remote rehabilitation"
#2 Method "Clinical trial"
#3 Published Since: 2000
#4 #1 AND #2 AND #3

#1 Abstract & Title: "Telehealth"
#2 Method "Clinical trial"
#3 Published Since: 2000
#4 #1 AND #2 AND #3

#1 Abstract & Title: "Telemedicine"
#2 Method "Clinical trial"
#3 Published Since: 2000
#4 #1 AND #2 AND #3

#1 Abstract & Title: "mobile health"
#2 Method "Clinical trial"
#3 Published Since: 2000
#4 #1 AND #2 AND #3

#1 Abstract & Title: "mhealth"
#2 Method "Clinical trial"
#3 Published Since: 2000
#4 #1 AND #2 AND #3

#1 Abstract & Title: "ehealth"
#2 Method "Clinical trial"
#3 Published Since: 2000
#4 #1 AND #2 AND #3

4. Search terms and search strategies in EBSCO

#1 Title Abstract Keyword: “Real-time telerehabilitation”
#2 Title Abstract Keyword: “Physical therapy”
#3 Publication Type: “Randomized control trial”
#1 AND #2 AND #3

#1 Title Abstract Keyword: “Real-time internet based”
#2 Title Abstract Keyword: “Physical therapy”
#3 Publication Type: “Randomized control trial”
#4 #1 AND #2 AND #3

#1 Title Abstract Keyword: “Telerehabilitation”
#2 Title Abstract Keyword: “Physical therapy”
#3 Publication Type: “Randomized control trial”
#4 #1 AND #2 AND #3

#1 Title Abstract Keyword: "Internet based"
#2 Title Abstract Keyword: “Physical therapy”
#3 Publication Type: “Randomized control trial”
#4 #1 AND #2 AND #3

#1 Title Abstract Keyword: "Remote rehabilitation"
#2 Title Abstract Keyword: “Physical therapy”
#3 Publication Type: “Randomized control trial”
#4 #1 AND #2 AND #3

#1 Title Abstract Keyword: "Telehealth"
#2 Title Abstract Keyword: “Physical therapy”
#3 Publication Type: “Randomized control trial”
#4 #1 AND #2 AND #3

#1 Title Abstract Keyword: "Telemedicine"
#2 Title Abstract Keyword: “Physical therapy”
#3 Publication Type: “Randomized control trial”
#4 #1 AND #2 AND #3

#1 Title Abstract Keyword: "Mobile health"
#2 Title Abstract Keyword: “Physical therapy”

#3 Publication Type: “Randomized control trial”
#4 #1 AND #2 AND #3

#1 Title Abstract Keyword: "Mhealth"
#2 Title Abstract Keyword: “Physical therapy”
#3 Publication Type: “Randomized control trial”
#4 #1 AND #2 AND #3

#1 Title Abstract Keyword: "ehealth"
#2 Title Abstract Keyword: “Physical therapy”
#3 Publication Type: “Randomized control trial”
#4 #1 AND #2 AND #3

5. Search terms and search strategies in ClinicalKey

#1 "Telerehabilitation"
#2 “Exercise”
#3 #1 AND #2

#1 "Telerehabilitation"
#2 “Training”
#3 #1 AND #2

#1 "Internet based"
#2 “Exercise”
#3 #1 AND #2

#1 "Internet based"
#2 “Training”
#3 #1 AND #2

#1 "Remote rehabilitation"
#2 “Exercise”
#3 #1 AND #2

#1 "Remote rehabilitation"
#2 “Training”
#3 #1 AND #2

#1 "Telehealth"
#2 “Exercise”

#3 #1 AND #2

#1 "Telehealth"
#2 “Training”
#3 #1 AND #2

#1 "TeleMedicine"
#2 “Exercise”
#3 #1 AND #2

#1 "TeleMedicine"
#2 “Training”
#3 #1 AND #2

#1 "Mobile Health"
#2 “Exercise”
#3 #1 AND #2

#1 " Mobile Health "
#2 “Training”
#3 #1 AND #2

#1 "MHealth"
#2 “Exercise”
#3 #1 AND #2

#1 " MHealth "
#2 “Training”
#3 #1 AND #2

#1 "EHealth"
#2 “Exercise”
#3 #1 AND #2

#1 " EHealth "
#2 “Training”
#3 #1 AND #2

6. Search terms and search strategies in ProQuest

#1(Real-time Telerehabilitation)
"Realtime Telerehabilitation"[Title/Abstract] OR "Realtime internet based"[Title/Abstract] OR "Telerehabilitation"[Title/Abstract] OR "internet based intervention"[Title/Abstract]

OR "Remote rehabilitation"[Title/Abstract] OR "Telehealth"[Title/Abstract] OR "Telemedicine"[Title/Abstract] OR "mobile health"[Title/Abstract] OR "mhealth"[Title/Abstract] OR "ehealth"[Title/Abstract] OR "social network service"[Title/Abstract]

#2(Physical Therapy)
"Physical Therapy"[All Fields] OR "Physiotherapist"[All Fields]

#3(Aging)
“Aging”[All Fields] OR “Senior”[All Fields] OR “Elderly”[All Fields] OR “Old adults”[All Fields]
#1 AND #2 AND #3
