# Supplementary material for: Real-Time Telerehabilitation in Older Adults With Musculoskeletal Conditions: Systematic Review and Meta-analysis
Source: JMIR Rehabil Assist Technol. 2022 Sep 1;9(3):e36028. doi: 10.2196/36028 (PMC9478822; doi:10.2196/36028)
Supplement: Multimedia Appendix 2 [file rehab_v9i3e36028_app2.pdf]

Table S1

**Author (Year)**	**Participants' ICT skills/ experience**	**Supervision by other than health professionals**	**Safety measure**	**Speed of internet**	**Other**
An et al, 2021	Familiarity with smartphone apps	None	None	None	No vision and hearing problems
Doricon-Cadrin et al, 2020	Instructions for use internet-based telecommunication mobile application	None	None	High-speed internet	None
Fernando et al, 2018	None	Caregivers assist the patient	Pain and fatigue scores (graduated from 0 to 10)	None	No problems with vision
Hong et al, 2017	None	None	The Rating of Perceived Exertion (RPE)	10 Mbps.	The participants were appropriately trained and experienced enough to ensure compliance and safety
Prvu Bettger et al, 2020	None	None	Patient-reported falls, pain, and hospital readmissions	None	None
Russell et al, 2003	None	None	None	18 kbps.	Wireless headphones were used to enable the subject to move about the room during the rehabilitation session
Russell et al, 2011	None	None	None	18 kbps.	None
Sparrow et al, 2011	None	None	The Rating of Perceived Exertion (RPE)	None	None
Tousignant et al, 2011	None	Family member or friend who had received prior training in the use of the technology that had been installed	Someone was to be at the participant home during tele-treatment	512 kbps.	None
Tousignant et al, 2015	None	None	None	512 kbps.	The system was mounted over a 20-inch LCD screen
